# Supplementary material for: Open Questions in Cold Atmospheric Plasma Treatment in Head and Neck Cancer: A Systematic Review
Source: Int J Mol Sci. 2022 Sep 6;23(18):10238. doi: 10.3390/ijms231810238 (PMC9498988; doi:10.3390/ijms231810238)
Supplement: Supplementary file 1 [file ijms-23-10238-s001.zip › ijms-1859565-supplementary.pdf]

Table S1 Search strategy input for PubMed, Web of Science and Scopus

DATABASES	SEARCH STRING
**PubMed:**	(cold atmospheric plasma OR cold plasma OR low temperature plasma OR kinpen med OR non-thermal atmospheric pressure plasma OR non thermal atmospheric pressure plasma OR cold physical plasma OR plasma medicine OR CAP OR plasma activated medium OR cold atmospheric-pressure plasma OR plasma activated liquid OR cold argon plasma) AND (head and neck tumors OR head and neck squamous cell carcinoma OR head and neck cancer OR head & neck cancer OR oral squamous cell carcinoma OR oral cancer OR oral squamous cell cancer OR OSCC OR oral oncology OR oral squamous cancer cell OR oral cavity squamous cell carcinoma) AND (therapy* or treat* OR application or attenuation OR efficacy OR action OR effect* OR target* OR response)
**Web of Science:**	Set for search in all Databases by Topic under Documents (cold atmospheric plasma OR cold plasma OR low temperature plasma OR kinpen med OR non-thermal atmospheric pressure plasma OR non thermal atmospheric pressure plasma OR cold physical plasma OR plasma medicine OR CAP OR plasma activated medium OR cold atmospheric-pressure plasma OR plasma activated liquid OR cold argon plasma) AND (head and neck tumors OR head and neck squamous cell carcinoma OR head and neck cancer OR head & neck cancer OR oral squamous cell carcinoma OR oral cancer OR oral squamous cell cancer OR OSCC OR oral oncology OR oral squamous cancer cell OR oral cavity squamous cell carcinoma) AND (therapy* or treat* OR application or attenuation OR efficacy OR action OR effect* OR target* OR response)<br>In addition, Refined by NOT Document Types: Abstract and Review Article
**Scopus:**	(ALL ("cold atmospheric plasma") AND ALL ("head and neck cancer"))

Table S2 Risk of Bias assessment in the included *in vitro* studies

Author/year	Condition of cell culture before treatment	Condition of cell culture during the treatment	Description of methodology to evaluate outcomes	Case control description	Multiple experiments performed	Description of plasma devices and settings
Choi B.B. et al. 2012[37]	A	A	A	A	A	A
Han X. et al. 2013[23]	I	A	A	A	A	A
Chang J.W. et al. 2014[36]	A	A	A	A	A	A
Guerrero-Preston R. et al. 2014[5]	A	A	A	A	I	A
Kang S.U. et al. 2014[73]	A	A	A	A	I	A
Kim S.Y. et al. 2015[74]	A	A	A	A	A	A
Welz C. et al. 2015[82]	A	A	A	A	A	A
Lee J.H. et al. 2016[54]	A	A	A	A	A	A
Chauvin J. et al. 2018[6]	A	A	A	A	A	A
Hasse S. et al. 2019[72]	A	A	A	A	A	A
Sato K. et al. 2019[80]	A	A	A	A	A	A
Han X. et al. 2020[71]	A	A	A	A	I	A
Lee C.M. et al. 2020[38]	A	A	A	A	A	A
Ramireddy L. et al. 2020[79]	A	A	A	A	A	A
Lin A. et al. 2021[76]	A	A	A	A	A	A
Oh C. et al. 2021[77]	A	A	A	A	A	A
Park J. et al. 2021[78]	A	A	A	A	A	A
Sklias K. et al. 2021[81]	A	A	A	A	A	A
Wu C.Y. et al. 2021[83]	A	A	A	A	A	A

Table S3 Risk of Bias assessment in the included *in vitro* studies

Author/year	Sequence generation	Baseline characteristics	Allocation concealment	Random housing	Blinding	Random outcome assessment	Blinding	Incomplete outcome data	Selective outcome reporting	Other sources of bias
Kang S.U. et al. 2014[73]	A	A	N/A	N/A	N/A	N/A	A	I	A	N/A
Kim S.Y. et al 2015[74]	A	A	N/A	N/A	N/A	N/A	I	I	A	N/A
Oh C. et al 2021[77]	A	A	N/A	N/A	N/A	N/A	I	A	A	N/A

Table S4 Risk of Bias assessment in the included *in vivo* studies with a case series study design according to Murad's checklist

Author/year	Selection	Ascertainment of Exposure	Ascertainment of Outcome	Causality - alternative causes	Causality - follow-up	Reporting
Metelmann H.R. et al. 2015[85]	I	A	A	A	A	A
Metelmann H.R. et al. 2018[15]	A	A	A	A	A	A
Schuster M. et al. 2018[87]	I	A	A	A	A	A

Table S5 Risk of Bias assessment according to NewCastle-Ottawa tool for human in vivo case-control study

Author/year	Adequacy of case definition	Representativeness of the cases	Selection of controls	Definition of controls	Comparability cases/controls	Ascertainment of exposure	Same method of ascertainment	Nonreponse rate
Dai X. et al. 2020[84]	A	A	A	A	A	A	A	N/A

Table S6 Risk of Bias assessment according to NewCastle-Ottawa tool for human in vivo cohort study

Author/year	Representativeness of exposed cohor	Selection of non-exposed cohort	Ascertainment of exposure	Demonstration that outcome of interest was not present at start of study	Comparability of cohorts on the basis of the design or analysis	Assessment of outcome	Follow-up length	Loss to follow-up rate
Schuster M. et al. 2016[87]	I	I	A	A	A	A	A	A
